# Bis(2,4,6-triamino-1,3,5-triazin-1-ium) bis­(4-hydroxy­pyridine-2,6-carboxyl­ato)­cuprate(II) hexa­hydrate

**DOI:** 10.1107/S160053680802566X

**Published:** 2008-08-16

**Authors:** Manuela Ramos Silva, Elham Motyeian, Hossein Aghabozorg, Mohammad Ghadermazi

**Affiliations:** a CEMDRX, Physics Department, University of Coimbra, P-3004-516 Coimbra, Portugal; b Department of Chemistry, Faculty of Science, Payame Noor University, Qom Center, Qom, Iran; c Faculty of Chemistry, Tarbiat Moallem University, 49 Mofateh Avenue, 15614 Tehran, Iran; d Department of Chemistry, Faculty of Science, University of Kurdistan, Sanandaj, Iran

## Abstract

In the title compound, (C_3_H_7_N_6_)_2_[Cu(C_7_H_3_NO_5_)_2_]·6H_2_O, the coordination geometry of the Cu^II^ atom can be described as distorted octa­hedral. The equatorial plane is defined by four O atoms from two 4-hydroxy­pyridine-2,6-dicarboxyl­ate ligands. The axial positions are occupied by the N atoms of the same ligands. There is an extensive three-dimensional hydrogen-bond network reinforcing crystal cohesion.

## Related literature

For related literature, see: Aghabozorg, Motyeian, Attar Gharamaleki *et al.* (2008 ▶); Aghabozorg, Motyeian, Soleimannejad *et al.* (2008 ▶); Aghabozorg, Saadaty *et al.* (2008 ▶).
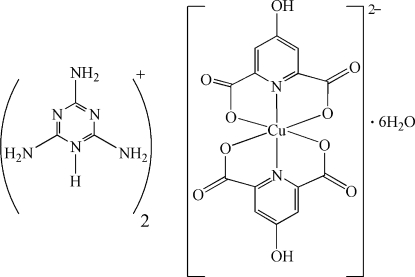


## Experimental

### 

#### Crystal data

(C_3_H_7_N_6_)_2_[Cu(C_7_H_3_NO_5_)_2_]·6H_2_O
*M* *_r_* = 788.14
Monoclinic, 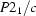

*a* = 11.2894 (3) Å
*b* = 37.7699 (12) Å
*c* = 7.3414 (2) Å
β = 94.016 (2)°
*V* = 3122.68 (15) Å^3^
*Z* = 4
Mo *K*α radiation
μ = 0.80 mm^−1^
*T* = 293 (2) K
0.28 × 0.20 × 0.10 mm

#### Data collection

Bruker APEX CCD area-detector diffractometer
Absorption correction: multi-scan (*SADABS*; Sheldrick, 2000 ▶) *T*_min_ = 0.714, *T*_max_ = 0.919
22082 measured reflections
7390 independent reflections
5112 reflections with *I* > 2σ(*I*)
*R*_int_ = 0.054

#### Refinement

*R*[*F* ^2^ > 2σ(*F* ^2^)] = 0.044
*wR*(*F* ^2^) = 0.116
*S* = 1.02
7390 reflections
508 parameters
13 restraints
H atoms treated by a mixture of independent and constrained refinement
Δρ_max_ = 0.44 e Å^−3^
Δρ_min_ = −0.46 e Å^−3^

### 

Data collection: *SMART* (Bruker, 2003 ▶); cell refinement: *SAINT* (Bruker, 2003 ▶); data reduction: *SAINT*; program(s) used to solve structure: *SHELXS97* (Sheldrick, 2008 ▶); program(s) used to refine structure: *SHELXL97* (Sheldrick, 2008 ▶); molecular graphics: *ORTEPII* (Johnson, 1976 ▶); software used to prepare material for publication: *SHELXL97*.

## Supplementary Material

Crystal structure: contains datablocks global, I. DOI: 10.1107/S160053680802566X/bt2763sup1.cif

Structure factors: contains datablocks I. DOI: 10.1107/S160053680802566X/bt2763Isup2.hkl

Additional supplementary materials:  crystallographic information; 3D view; checkCIF report

## Figures and Tables

**Table 1:** Hydrogen-bond geometry (Å, °)

*D*—H⋯*A*	*D*—H	H⋯*A*	*D*⋯*A*	*D*—H⋯*A*
O1*A*—H1*A*⋯O7^i^	0.74 (3)	1.84 (3)	2.573 (3)	169 (4)
O1*B*—H1*B*⋯O6^ii^	0.76 (4)	1.83 (4)	2.579 (3)	173 (4)
N5*A*—H5*A*⋯O10^ii^	0.77 (2)	2.06 (3)	2.787 (3)	159 (3)
N6*A*—H7⋯O4*B*^iii^	0.86	2.13	2.980 (3)	173
N6*A*—H8⋯O11	0.86	2.13	2.962 (4)	162
N7*A*—H11⋯O1*B*^iii^	0.86	2.25	3.106 (3)	172
N7*A*—H12⋯O9^iv^	0.86	2.11	2.910 (4)	155
N8*A*—H9⋯N3*A*^v^	0.86	2.11	2.973 (4)	177
N8*A*—H10⋯O10^ii^	0.86	2.25	2.986 (4)	144
N8*A*—H10⋯O11^v^	0.86	2.56	3.202 (4)	133
N5*B*—H5*B*⋯O2*A*^vi^	0.78 (2)	1.96 (3)	2.698 (3)	157 (3)
N6*B*—H5⋯O1*A*^i^	0.86	2.28	3.131 (3)	170
N6*B*—H6⋯O9	0.86	2.38	2.905 (3)	120
N7*B*—H1⋯O5*B*^vii^	0.86	2.24	3.021 (3)	151
N7*B*—H2⋯O2*A*^vi^	0.86	2.11	2.852 (3)	144
N8*B*—H3⋯O4*A*^i^	0.86	2.10	2.930 (3)	163
N8*B*—H4⋯O8^vi^	0.86	2.01	2.857 (3)	169
O6—H61⋯O5*A*	0.83 (2)	1.87 (2)	2.706 (3)	177 (4)
O6—H62⋯O2*B*^viii^	0.81 (2)	1.97 (2)	2.756 (3)	163 (4)
O7—H71⋯O3*B*^viii^	0.82 (2)	1.94 (2)	2.741 (3)	163 (4)
O7—H72⋯O5*B*	0.82 (2)	1.95 (2)	2.766 (3)	171 (4)
O8—H81⋯O4*B*^iii^	0.86 (2)	1.86 (2)	2.714 (3)	173 (4)
O8—H82⋯O3*A*	0.83 (2)	1.90 (2)	2.701 (3)	162 (4)
O9—H91⋯O3*B*	0.88 (2)	1.97 (2)	2.839 (3)	172 (4)
O9—H92⋯O4*A*^iii^	0.88 (2)	2.13 (2)	3.005 (3)	176 (4)
O10—H101⋯O8^ix^	0.84 (4)	2.24 (3)	3.030 (4)	157 (5)
O10—H102⋯O4*A*	0.89 (4)	2.39 (5)	2.695 (3)	101 (4)
O11—H111⋯O10^x^	0.85 (2)	2.44 (4)	3.196 (5)	149 (7)
O11—H112⋯N4*B*^x^	0.83 (6)	2.53 (7)	2.996 (4)	116 (6)
O11—H112⋯O8	0.83 (6)	2.60 (8)	3.090 (5)	118 (7)

Symmetry codes: (i) 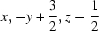; (ii) 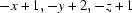; (iii) 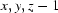; (iv) 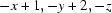; (v) 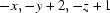; (vi) 
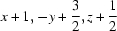
; (vii) 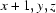; (viii) 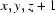; (ix) 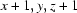; (x) 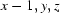.

**Fig. 1.**
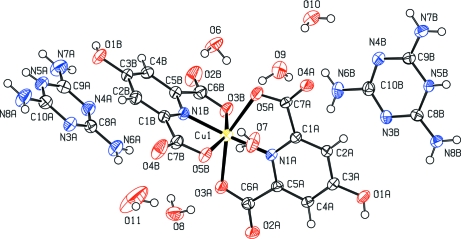
ORTEPII (Johnson, 1976) plot of the title compound. Displacement ellipsoids are drawn at the 50% level.

**Fig. 2.**
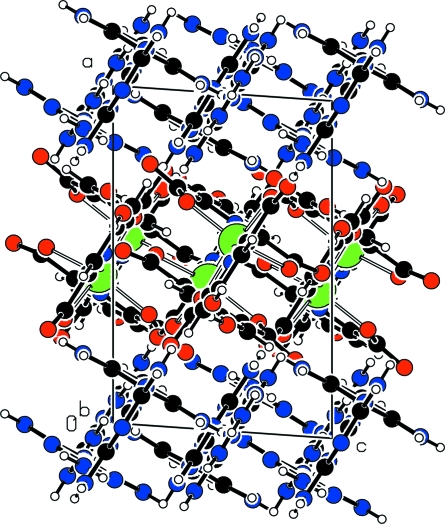
Packing diagram of the title compound. Water molecules were excluded for clarity.

**Fig. 3.**
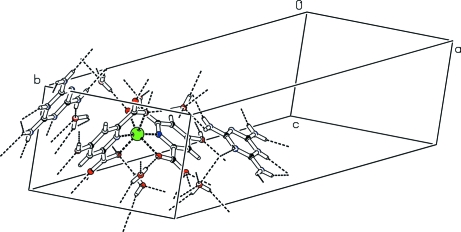
A part of the extensive three-dimensional H-bond network. H-bonds are depicted as dashed lines.

## supplementary crystallographic information

### Comment

Following our research on the synthesis of proton transfer compounds that can function as suitable ligands in the synthesis of metal complexes (Aghabozorg, Motyeian, Attar Gharamaleki *et al.*, 2008; Aghabozorg, Motyeian, Soleimannejad *et al.*, 2008; Aghabozorg, Saadaty *et al.*, 2008), we have obtained the title compound dimelaminium bis(4-hydroxypyridine-2,6-carboxylato)cuprate(II) hexahydrated. 4-hydroxypyridine-2,6-carboxylic acid (hypydcH2) was chosen as a proton donor and melamine (tata) as the proton acceptor.

The asymmetric unit of (I) consists of two melaminium (tataH) residues protonated at one ring N atom, two (hypydc) residues coordinating a Cu^II^ ion and six water molecules (Fig. 1). The melaminium cations are essentially planar with the weighted average absolute torsion angle equal to 0.67 (23) for ring A and 1.20 (33)° for ring B. Both rings exhibit a significant distortion from the ideal hexagonal form. The internal C—N—C angle of the protonated N atom (N5A, N5B) is significantly larger than the other two ring C—N—C angles (Table 1). The angle between the least-squares plane of the two independent cations is 87.97 (12)°. The anions also assemble perpendicularly to each other. The angle between the mean planes of the two independent pyridil rings is 89.51 (12)°. Thus the molecules form a square grid with channels along the *b* axis (Fig. 2). The Cu^II^ ion is coordinated octahedrally by two ligands of (hypydc). The N atoms of the two independent anions occupy the axial positions while four oxygen atoms form the equatorial plane. There is an extensive network of hydrogen bonds proportionated by the large amount of water molecules. All the water molecules share their hydrogen atoms with another strong acceptor (N,*O*). The (hypydc) anions have similar H-bonds, but the two independent melaminium cations have different roles in the web of H-bonds. While B molecules only establish H-bonds to neighbouring water or (hypydc) molecules, the A molecules are also joined in dimers (Fig.3, Table 2).

### Experimental

The proton transfer compound, (tata)_2_(hypydc), was prepared by the reaction of 4-hydroxypyridinee-2,6-dicarboxylic acid, hypydcH_2_, with melamine, (tata). The reaction between Cu(NO_3_)_2_.6H_2_O (143 mg, 0.5 mmol) in water (20 ml) and proton transfer compound, (phenH)_2_(hypydc) (253 mg, 1.0 mmol) in water (20 ml), in a 1:2 molar ratio was carried out and a blue crystalline compound was obtained by the slow evaporation of the solvent at room temperature.

### Refinement

All H-atoms could be located in difference Fourier maps. The H atoms of water molecules were refined with an O—H distance restraint of 0.85 (2) Å and *U*_iso_(H) = 1.5*U*_eq_(O). Short contacts between the H atoms of the water O10 and neighbouring H atoms are observed at the final refinement, an indication that these H atoms are probably disordered. The coordinates of the H atoms of the hydroxyl groups were freely refined with *U*_iso_(H) = 1.5*U*_eq_(O), the H atoms bonded to the N atoms of the melaminium rings were restrained to have equal N—H distances and *U*_iso_(H) = 1.2*U*_eq_(N). The remaining H atoms were placed at calculated positions and refined as riding on their parent atoms with *U*_iso_(H) = 1.2*U*_eq_(N,*C*)

### Crystal data

**Table:**

(C_3_H_7_N_6_)_2_[Cu(C_7_H_3_N_1_O_5_)_2_]·6H_2_O	*F*_000_ = 1628
*M**_r_* = 788.14	*D*_x_ = 1.676 Mg m^−^^3^
Monoclinic, *P*2_1_/*c*	Mo *K*α radiation λ = 0.71073 Å
*a* = 11.2894 (3) Å	Cell parameters from 4074 reflections
*b* = 37.7699 (12) Å	θ = 2.4–24.5º
*c* = 7.3414 (2) Å	µ = 0.80 mm^−^^1^
β = 94.016 (2)º	*T* = 293 (2) K
*V* = 3122.68 (15) Å^3^	Prism, green
*Z* = 4	0.28 × 0.20 × 0.10 mm

### Data collection

**Table:**

Bruker APEX CCD area-detector diffractometer	7390 independent reflections
Radiation source: fine-focus sealed tube	5112 reflections with *I* > 2σ(*I*)
Monochromator: graphite	*R*_int_ = 0.054
*T* = 293(2) K	θ_max_ = 28.3º
φ and ω scans	θ_min_ = 1.8º
Absorption correction: multi-scan(SADABS; Sheldrick, 2000)	*h* = −15→14
*T*_min_ = 0.714, *T*_max_ = 0.919	*k* = −50→50
22082 measured reflections	*l* = −7→9

### Refinement

**Table:**

Refinement on *F*^2^	Secondary atom site location: difference Fourier map
Least-squares matrix: full	Hydrogen site location: inferred from neighbouring sites
*R*[*F*^2^ > 2σ(*F*^2^)] = 0.044	H atoms treated by a mixture of independent and constrained refinement
*wR*(*F*^2^) = 0.116	*w* = 1/[σ^2^(*F*_o_^2^) + (0.0572*P*)^2^ + 0.0192*P*] where *P* = (*F*_o_^2^ + 2*F*_c_^2^)/3
*S* = 1.03	(Δ/σ)_max_ = 0.014
7390 reflections	Δρ_max_ = 0.44 e Å^−^^3^
508 parameters	Δρ_min_ = −0.46 e Å^−^^3^
13 restraints	Extinction correction: none
Primary atom site location: structure-invariant direct methods	

### Special details

**Table:**

Geometry. All e.s.d.'s (except the e.s.d. in the dihedral angle between two l.s. planes) are estimated using the full covariance matrix. The cell e.s.d.'s are taken into account individually in the estimation of e.s.d.'s in distances, angles and torsion angles; correlations between e.s.d.'s in cell parameters are only used when they are defined by crystal symmetry. An approximate (isotropic) treatment of cell e.s.d.'s is used for estimating e.s.d.'s involving l.s. planes.
Refinement. Refinement of *F*^2^ against ALL reflections. The weighted *R*-factor *wR* and goodness of fit *S* are based on *F*^2^, conventional *R*-factors *R* are based on *F*, with *F* set to zero for negative *F*^2^. The threshold expression of *F*^2^ > σ(*F*^2^) is used only for calculating *R*-factors(gt) *etc*. and is not relevant to the choice of reflections for refinement. *R*-factors based on *F*^2^ are statistically about twice as large as those based on *F*, and *R*- factors based on ALL data will be even larger.

### Fractional atomic coordinates and isotropic or equivalent isotropic displacement parameters (Å^2^)

**Table:**

	*x*	*y*	*z*	*U*_iso_*/*U*_eq_	
Cu1	0.43767 (3)	0.864573 (8)	0.43487 (5)	0.02540 (10)	
O1A	0.62393 (18)	0.71521 (5)	0.5449 (3)	0.0339 (5)	
H1A	0.584 (3)	0.7012 (9)	0.503 (5)	0.051*	
O1B	0.31432 (19)	1.01748 (5)	0.5212 (3)	0.0363 (5)	
H1B	0.335 (3)	1.0291 (9)	0.446 (5)	0.054*	
O2A	0.26377 (16)	0.77504 (5)	0.2033 (3)	0.0382 (5)	
O2B	0.5124 (2)	0.93825 (6)	0.0457 (3)	0.0532 (7)	
O3A	0.29856 (16)	0.83148 (5)	0.2762 (3)	0.0321 (4)	
O3B	0.50694 (17)	0.88676 (5)	0.1949 (3)	0.0339 (5)	
O4A	0.75786 (16)	0.83882 (5)	0.7359 (3)	0.0356 (5)	
O4B	0.2249 (2)	0.89919 (5)	0.8309 (3)	0.0524 (7)	
O5A	0.61471 (17)	0.87161 (5)	0.5943 (3)	0.0368 (5)	
O5B	0.32894 (17)	0.86291 (5)	0.6639 (3)	0.0313 (4)	
N1A	0.50099 (18)	0.81629 (5)	0.4577 (3)	0.0218 (4)	
N1B	0.38376 (18)	0.91274 (5)	0.4434 (3)	0.0235 (5)	
C1A	0.6033 (2)	0.80997 (6)	0.5575 (4)	0.0218 (5)	
C1B	0.3243 (2)	0.92336 (7)	0.5850 (4)	0.0255 (6)	
C2A	0.6461 (2)	0.77631 (6)	0.5867 (4)	0.0245 (6)	
H2A	0.7177	0.7726	0.6548	0.029*	
C2B	0.2994 (2)	0.95844 (7)	0.6138 (4)	0.0299 (6)	
H2B	0.2580	0.9655	0.7129	0.036*	
C3A	0.5813 (2)	0.74770 (6)	0.5132 (4)	0.0239 (5)	
C3B	0.3381 (2)	0.98330 (6)	0.4897 (4)	0.0272 (6)	
C4A	0.4744 (2)	0.75443 (7)	0.4131 (4)	0.0248 (6)	
H4A	0.4283	0.7358	0.3646	0.030*	
C4B	0.3979 (2)	0.97189 (7)	0.3412 (4)	0.0272 (6)	
H4B	0.4226	0.9880	0.2558	0.033*	
C5A	0.4376 (2)	0.78907 (6)	0.3866 (4)	0.0222 (5)	
C5B	0.4196 (2)	0.93624 (7)	0.3236 (4)	0.0249 (6)	
C6A	0.3237 (2)	0.79909 (7)	0.2795 (4)	0.0257 (6)	
C6B	0.4848 (2)	0.91968 (7)	0.1720 (4)	0.0303 (6)	
C7A	0.6650 (2)	0.84270 (6)	0.6363 (4)	0.0250 (6)	
C7B	0.2893 (2)	0.89329 (7)	0.7058 (4)	0.0305 (6)	
N3A	0.0763 (2)	0.99122 (6)	0.2789 (3)	0.0325 (5)	
N4A	0.1682 (2)	0.99282 (6)	−0.0069 (3)	0.0342 (6)	
N5A	0.1108 (2)	1.04478 (6)	0.1332 (4)	0.0341 (6)	
H5A	0.099 (3)	1.0648 (7)	0.133 (5)	0.041*	
N6A	0.1337 (2)	0.94103 (6)	0.1355 (4)	0.0390 (6)	
H7	0.1647	0.9304	0.0467	0.047*	
H8	0.1079	0.9289	0.2237	0.047*	
N7A	0.1989 (2)	1.04682 (7)	−0.1378 (4)	0.0460 (7)	
H11	0.2294	1.0367	−0.2284	0.055*	
H12	0.1934	1.0695	−0.1344	0.055*	
N8A	0.0229 (2)	1.04431 (7)	0.4041 (4)	0.0476 (7)	
H9	−0.0045	1.0334	0.4949	0.057*	
H10	0.0195	1.0670	0.3983	0.057*	
C8A	0.1257 (2)	0.97605 (7)	0.1365 (4)	0.0306 (6)	
C9A	0.1607 (2)	1.02764 (7)	−0.0048 (4)	0.0342 (7)	
C10A	0.0699 (2)	1.02625 (7)	0.2739 (4)	0.0336 (7)	
N3B	0.88638 (18)	0.76048 (6)	0.3504 (3)	0.0289 (5)	
N4B	1.0116 (2)	0.80888 (6)	0.4584 (3)	0.0321 (5)	
N5B	1.06806 (18)	0.75026 (6)	0.5151 (3)	0.0266 (5)	
H5B	1.114 (2)	0.7379 (7)	0.567 (4)	0.032*	
N6B	0.8337 (2)	0.81741 (6)	0.2915 (4)	0.0395 (6)	
H5	0.7703	0.8094	0.2338	0.047*	
H6	0.8458	0.8399	0.2990	0.047*	
N7B	1.1849 (2)	0.79554 (6)	0.6287 (3)	0.0347 (6)	
H1	1.2006	0.8177	0.6442	0.042*	
H2	1.2329	0.7798	0.6758	0.042*	
N8B	0.95084 (19)	0.70392 (6)	0.4094 (3)	0.0330 (6)	
H3	0.8879	0.6957	0.3518	0.040*	
H4	1.0036	0.6897	0.4577	0.040*	
C8B	0.9664 (2)	0.73824 (7)	0.4237 (4)	0.0255 (6)	
C9B	1.0871 (2)	0.78572 (7)	0.5325 (4)	0.0262 (6)	
C10B	0.9134 (2)	0.79486 (7)	0.3697 (4)	0.0286 (6)	
O6	0.6260 (2)	0.93858 (5)	0.7253 (3)	0.0433 (6)	
H61	0.620 (3)	0.9180 (6)	0.684 (5)	0.065*	
H62	0.580 (3)	0.9381 (10)	0.805 (4)	0.065*	
O7	0.4746 (2)	0.83354 (6)	0.9426 (3)	0.0472 (6)	
H71	0.469 (3)	0.8491 (8)	1.020 (4)	0.071*	
H72	0.429 (3)	0.8401 (10)	0.857 (4)	0.071*	
O8	0.1197 (2)	0.84896 (6)	0.0289 (4)	0.0508 (6)	
H81	0.150 (3)	0.8641 (9)	−0.041 (5)	0.076*	
H82	0.168 (3)	0.8391 (10)	0.104 (5)	0.076*	
O9	0.7422 (2)	0.87866 (6)	0.0878 (4)	0.0527 (6)	
H91	0.672 (2)	0.8828 (11)	0.127 (6)	0.079*	
H92	0.750 (4)	0.8675 (10)	−0.015 (4)	0.079*	
O10	0.9382 (2)	0.88564 (6)	0.7704 (5)	0.0662 (8)	
H101	0.984 (4)	0.8704 (10)	0.821 (6)	0.099*	
H102	0.894 (4)	0.8820 (13)	0.864 (5)	0.099*	
O11	0.0445 (5)	0.88589 (8)	0.3782 (7)	0.1234 (17)	
H111	0.030 (7)	0.878 (2)	0.481 (5)	0.185*	
H112	0.088 (6)	0.8685 (13)	0.362 (12)	0.185*	

### Atomic displacement parameters (Å^2^)

**Table:**

	*U*^11^	*U*^22^	*U*^33^	*U*^12^	*U*^13^	*U*^23^
Cu1	0.02778 (17)	0.01879 (16)	0.0296 (2)	0.00051 (12)	0.00170 (13)	−0.00093 (13)
O1A	0.0396 (12)	0.0164 (9)	0.0439 (13)	0.0007 (8)	−0.0101 (9)	−0.0015 (8)
O1B	0.0498 (12)	0.0172 (10)	0.0435 (14)	0.0027 (8)	0.0149 (10)	0.0008 (8)
O2A	0.0343 (11)	0.0276 (10)	0.0501 (14)	−0.0027 (8)	−0.0156 (10)	−0.0076 (9)
O2B	0.0761 (16)	0.0373 (12)	0.0501 (16)	0.0082 (11)	0.0337 (13)	0.0108 (11)
O3A	0.0336 (10)	0.0225 (9)	0.0390 (13)	0.0026 (8)	−0.0065 (9)	0.0010 (8)
O3B	0.0428 (11)	0.0214 (10)	0.0382 (13)	0.0037 (8)	0.0069 (10)	−0.0046 (8)
O4A	0.0291 (10)	0.0250 (10)	0.0502 (14)	−0.0024 (8)	−0.0150 (9)	−0.0037 (9)
O4B	0.0794 (16)	0.0281 (11)	0.0549 (16)	0.0079 (10)	0.0410 (14)	0.0079 (10)
O5A	0.0377 (11)	0.0191 (9)	0.0518 (14)	0.0035 (8)	−0.0100 (10)	−0.0060 (9)
O5B	0.0417 (11)	0.0202 (9)	0.0323 (12)	0.0045 (8)	0.0037 (9)	0.0014 (8)
N1A	0.0245 (11)	0.0182 (10)	0.0227 (12)	0.0002 (8)	0.0017 (9)	−0.0011 (8)
N1B	0.0254 (11)	0.0198 (10)	0.0254 (13)	0.0028 (8)	0.0027 (9)	0.0027 (9)
C1A	0.0229 (12)	0.0201 (12)	0.0224 (15)	0.0000 (9)	0.0015 (10)	−0.0030 (10)
C1B	0.0246 (12)	0.0234 (13)	0.0287 (16)	0.0009 (10)	0.0038 (11)	0.0015 (11)
C2A	0.0220 (12)	0.0212 (12)	0.0302 (16)	0.0006 (9)	0.0000 (11)	−0.0023 (11)
C2B	0.0301 (14)	0.0250 (13)	0.0351 (17)	0.0031 (11)	0.0067 (12)	0.0001 (12)
C3A	0.0292 (13)	0.0183 (12)	0.0245 (15)	0.0009 (10)	0.0030 (11)	−0.0001 (10)
C3B	0.0262 (13)	0.0193 (12)	0.0360 (17)	0.0021 (10)	0.0019 (12)	0.0022 (11)
C4A	0.0262 (13)	0.0190 (12)	0.0289 (16)	−0.0027 (10)	−0.0002 (11)	−0.0036 (10)
C4B	0.0276 (13)	0.0224 (13)	0.0318 (17)	−0.0022 (10)	0.0037 (12)	0.0043 (11)
C5A	0.0229 (12)	0.0206 (12)	0.0232 (15)	−0.0019 (9)	0.0012 (11)	−0.0022 (10)
C5B	0.0245 (13)	0.0226 (13)	0.0275 (16)	0.0005 (10)	0.0017 (11)	0.0003 (11)
C6A	0.0271 (13)	0.0246 (13)	0.0253 (16)	−0.0013 (10)	0.0012 (11)	0.0010 (11)
C6B	0.0327 (14)	0.0274 (14)	0.0311 (17)	−0.0013 (11)	0.0051 (12)	−0.0003 (12)
C7A	0.0260 (13)	0.0176 (12)	0.0314 (16)	−0.0025 (10)	0.0029 (11)	−0.0035 (10)
C7B	0.0362 (15)	0.0222 (13)	0.0335 (18)	0.0020 (11)	0.0044 (13)	0.0031 (11)
N3A	0.0371 (13)	0.0240 (12)	0.0367 (15)	0.0024 (10)	0.0031 (11)	−0.0027 (10)
N4A	0.0379 (13)	0.0278 (12)	0.0371 (16)	0.0018 (10)	0.0047 (11)	−0.0040 (10)
N5A	0.0375 (13)	0.0211 (11)	0.0445 (16)	−0.0004 (10)	0.0073 (12)	−0.0027 (11)
N6A	0.0495 (15)	0.0254 (12)	0.0430 (17)	0.0028 (10)	0.0098 (13)	−0.0018 (11)
N7A	0.0574 (17)	0.0341 (14)	0.0485 (18)	−0.0038 (12)	0.0171 (14)	−0.0009 (13)
N8A	0.0653 (18)	0.0277 (13)	0.0522 (19)	0.0046 (12)	0.0208 (15)	−0.0060 (12)
C8A	0.0267 (14)	0.0267 (14)	0.0375 (18)	0.0016 (11)	−0.0036 (12)	−0.0017 (12)
C9A	0.0279 (14)	0.0334 (15)	0.0410 (19)	−0.0029 (11)	0.0008 (13)	−0.0005 (13)
C10A	0.0313 (14)	0.0299 (15)	0.0396 (19)	−0.0003 (11)	0.0022 (13)	−0.0051 (13)
N3B	0.0258 (11)	0.0269 (12)	0.0334 (14)	0.0040 (9)	−0.0026 (10)	0.0041 (10)
N4B	0.0325 (12)	0.0230 (11)	0.0409 (15)	0.0012 (9)	0.0030 (11)	0.0018 (10)
N5B	0.0228 (11)	0.0196 (11)	0.0364 (15)	0.0029 (8)	−0.0044 (10)	0.0044 (9)
N6B	0.0369 (13)	0.0314 (13)	0.0493 (17)	0.0109 (10)	−0.0036 (12)	0.0092 (12)
N7B	0.0344 (13)	0.0228 (12)	0.0458 (17)	−0.0043 (10)	−0.0054 (11)	−0.0004 (10)
N8B	0.0282 (12)	0.0222 (11)	0.0469 (17)	−0.0009 (9)	−0.0087 (11)	0.0000 (10)
C8B	0.0223 (12)	0.0265 (14)	0.0280 (16)	−0.0016 (10)	0.0032 (11)	0.0007 (11)
C9B	0.0260 (13)	0.0231 (13)	0.0296 (16)	−0.0020 (10)	0.0036 (11)	0.0012 (11)
C10B	0.0296 (14)	0.0273 (14)	0.0293 (16)	0.0039 (11)	0.0041 (12)	0.0043 (11)
O6	0.0679 (16)	0.0225 (10)	0.0415 (15)	−0.0086 (10)	0.0179 (11)	−0.0008 (10)
O7	0.0685 (16)	0.0315 (12)	0.0393 (15)	0.0207 (11)	−0.0129 (12)	−0.0065 (10)
O8	0.0402 (13)	0.0409 (13)	0.0688 (19)	−0.0105 (10)	−0.0137 (12)	0.0193 (12)
O9	0.0477 (14)	0.0393 (13)	0.071 (2)	0.0071 (11)	0.0057 (13)	0.0026 (12)
O10	0.0493 (16)	0.0298 (13)	0.119 (3)	−0.0038 (11)	−0.0005 (16)	0.0053 (14)
O11	0.201 (5)	0.0406 (18)	0.139 (4)	−0.011 (2)	0.086 (3)	0.004 (2)

### Geometric parameters (Å, °)

**Table:**

Cu1—N1B	1.921 (2)	N4A—C8A	1.346 (4)
Cu1—N1A	1.962 (2)	N5A—C10A	1.355 (4)
Cu1—O3B	2.147 (2)	N5A—C9A	1.357 (4)
Cu1—O5B	2.1508 (19)	N5A—H5A	0.77 (2)
Cu1—O5A	2.260 (2)	N6A—C8A	1.326 (3)
Cu1—O3A	2.2648 (19)	N6A—H7	0.8600
O1A—C3A	1.333 (3)	N6A—H8	0.8600
O1A—H1A	0.74 (3)	N7A—C9A	1.313 (4)
O1B—C3B	1.342 (3)	N7A—H11	0.8600
O1B—H1B	0.76 (4)	N7A—H12	0.8600
O2A—C6A	1.242 (3)	N8A—C10A	1.316 (4)
O2B—C6B	1.221 (3)	N8A—H9	0.8600
O3A—C6A	1.256 (3)	N8A—H10	0.8600
O3B—C6B	1.277 (3)	N3B—C8B	1.321 (3)
O4A—C7A	1.244 (3)	N3B—C10B	1.339 (3)
O4B—C7B	1.231 (3)	N4B—C9B	1.312 (3)
O5A—C7A	1.259 (3)	N4B—C10B	1.353 (4)
O5B—C7B	1.277 (3)	N5B—C9B	1.361 (3)
N1A—C5A	1.338 (3)	N5B—C8B	1.365 (3)
N1A—C1A	1.345 (3)	N5B—H5B	0.78 (2)
N1B—C5B	1.332 (3)	N6B—C10B	1.339 (3)
N1B—C1B	1.338 (3)	N6B—H5	0.8600
C1A—C2A	1.372 (3)	N6B—H6	0.8600
C1A—C7A	1.514 (3)	N7B—C9B	1.322 (3)
C1B—C2B	1.374 (4)	N7B—H1	0.8600
C1B—C7B	1.510 (4)	N7B—H2	0.8600
C2A—C3A	1.393 (3)	N8B—C8B	1.311 (3)
C2A—H2A	0.9300	N8B—H3	0.8600
C2B—C3B	1.400 (4)	N8B—H4	0.8600
C2B—H2B	0.9300	O6—H61	0.834 (18)
C3A—C4A	1.391 (4)	O6—H62	0.808 (18)
C3B—C4B	1.390 (4)	O7—H71	0.821 (18)
C4A—C5A	1.382 (3)	O7—H72	0.821 (19)
C4A—H4A	0.9300	O8—H81	0.86 (4)
C4B—C5B	1.377 (3)	O8—H82	0.84 (4)
C4B—H4B	0.9300	O9—H91	0.877 (18)
C5A—C6A	1.507 (4)	O9—H92	0.876 (19)
C5B—C6B	1.512 (4)	O10—H101	0.84 (4)
N3A—C10A	1.325 (3)	O10—H102	0.89 (4)
N3A—C8A	1.347 (4)	O11—H111	0.85 (2)
N4A—C9A	1.318 (3)	O11—H112	0.83 (6)
			
N1B—Cu1—N1A	172.83 (10)	O2B—C6B—C5B	119.0 (2)
N1B—Cu1—O3B	77.96 (8)	O3B—C6B—C5B	114.0 (2)
N1A—Cu1—O3B	106.34 (8)	O4A—C7A—O5A	126.4 (2)
N1B—Cu1—O5B	78.63 (8)	O4A—C7A—C1A	118.3 (2)
N1A—Cu1—O5B	97.51 (8)	O5A—C7A—C1A	115.3 (2)
O3B—Cu1—O5B	155.97 (7)	O4B—C7B—O5B	125.3 (3)
N1B—Cu1—O5A	98.19 (8)	O4B—C7B—C1B	119.6 (2)
N1A—Cu1—O5A	76.19 (8)	O5B—C7B—C1B	115.1 (2)
O3B—Cu1—O5A	91.11 (8)	C10A—N3A—C8A	115.3 (2)
O5B—Cu1—O5A	97.53 (8)	C9A—N4A—C8A	115.7 (2)
N1B—Cu1—O3A	109.24 (8)	C10A—N5A—C9A	120.3 (2)
N1A—Cu1—O3A	76.48 (8)	C10A—N5A—H5A	116 (2)
O3B—Cu1—O3A	94.16 (7)	C9A—N5A—H5A	123 (2)
O5B—Cu1—O3A	88.44 (7)	C8A—N6A—H7	120.0
O5A—Cu1—O3A	152.56 (6)	C8A—N6A—H8	120.0
C3A—O1A—H1A	112 (3)	H7—N6A—H8	120.0
C3B—O1B—H1B	111 (3)	C9A—N7A—H11	120.0
C6A—O3A—Cu1	112.32 (16)	C9A—N7A—H12	120.0
C6B—O3B—Cu1	114.11 (17)	H11—N7A—H12	120.0
C7A—O5A—Cu1	113.08 (16)	C10A—N8A—H9	120.0
C7B—O5B—Cu1	113.01 (17)	C10A—N8A—H10	120.0
C5A—N1A—C1A	119.4 (2)	H9—N8A—H10	120.0
C5A—N1A—Cu1	119.96 (17)	N6A—C8A—N4A	115.9 (3)
C1A—N1A—Cu1	120.37 (16)	N6A—C8A—N3A	117.4 (3)
C5B—N1B—C1B	120.4 (2)	N4A—C8A—N3A	126.7 (2)
C5B—N1B—Cu1	119.86 (17)	N7A—C9A—N4A	121.1 (3)
C1B—N1B—Cu1	119.10 (17)	N7A—C9A—N5A	117.8 (3)
N1A—C1A—C2A	122.0 (2)	N4A—C9A—N5A	121.0 (3)
N1A—C1A—C7A	114.7 (2)	N8A—C10A—N3A	121.4 (3)
C2A—C1A—C7A	123.3 (2)	N8A—C10A—N5A	117.5 (3)
N1B—C1B—C2B	121.9 (2)	N3A—C10A—N5A	121.0 (3)
N1B—C1B—C7B	113.4 (2)	C8B—N3B—C10B	115.4 (2)
C2B—C1B—C7B	124.7 (2)	C9B—N4B—C10B	115.2 (2)
C1A—C2A—C3A	119.2 (2)	C9B—N5B—C8B	119.7 (2)
C1A—C2A—H2A	120.4	C9B—N5B—H5B	117 (2)
C3A—C2A—H2A	120.4	C8B—N5B—H5B	123 (2)
C1B—C2B—C3B	118.0 (2)	C10B—N6B—H5	120.0
C1B—C2B—H2B	121.0	C10B—N6B—H6	120.0
C3B—C2B—H2B	121.0	H5—N6B—H6	120.0
O1A—C3A—C4A	123.4 (2)	C9B—N7B—H1	120.0
O1A—C3A—C2A	118.2 (2)	C9B—N7B—H2	120.0
C4A—C3A—C2A	118.4 (2)	H1—N7B—H2	120.0
O1B—C3B—C4B	123.2 (2)	C8B—N8B—H3	120.0
O1B—C3B—C2B	117.2 (2)	C8B—N8B—H4	120.0
C4B—C3B—C2B	119.6 (2)	H3—N8B—H4	120.0
C5A—C4A—C3A	119.2 (2)	N8B—C8B—N3B	120.8 (2)
C5A—C4A—H4A	120.4	N8B—C8B—N5B	118.1 (2)
C3A—C4A—H4A	120.4	N3B—C8B—N5B	121.1 (2)
C5B—C4B—C3B	118.5 (2)	N4B—C9B—N7B	121.9 (2)
C5B—C4B—H4B	120.8	N4B—C9B—N5B	121.5 (2)
C3B—C4B—H4B	120.8	N7B—C9B—N5B	116.5 (2)
N1A—C5A—C4A	121.7 (2)	N3B—C10B—N6B	115.5 (3)
N1A—C5A—C6A	115.1 (2)	N3B—C10B—N4B	127.1 (2)
C4A—C5A—C6A	123.2 (2)	N6B—C10B—N4B	117.4 (2)
N1B—C5B—C4B	121.7 (2)	H61—O6—H62	101 (3)
N1B—C5B—C6B	113.4 (2)	H71—O7—H72	104 (4)
C4B—C5B—C6B	125.0 (2)	H81—O8—H82	116 (4)
O2A—C6A—O3A	126.0 (3)	H91—O9—H92	121 (4)
O2A—C6A—C5A	118.0 (2)	H101—O10—H102	85 (4)
O3A—C6A—C5A	116.0 (2)	H111—O11—H112	89 (6)
O2B—C6B—O3B	126.9 (3)		
			
N1B—Cu1—O3A—C6A	−174.06 (18)	C1A—N1A—C5A—C4A	0.1 (4)
N1A—Cu1—O3A—C6A	1.43 (18)	Cu1—N1A—C5A—C4A	−174.41 (19)
O3B—Cu1—O3A—C6A	107.24 (18)	C1A—N1A—C5A—C6A	179.2 (2)
O5B—Cu1—O3A—C6A	−96.68 (18)	Cu1—N1A—C5A—C6A	4.7 (3)
O5A—Cu1—O3A—C6A	6.7 (3)	C3A—C4A—C5A—N1A	−1.3 (4)
N1B—Cu1—O3B—C6B	1.61 (19)	C3A—C4A—C5A—C6A	179.7 (2)
N1A—Cu1—O3B—C6B	−172.46 (19)	C1B—N1B—C5B—C4B	0.4 (4)
O5B—Cu1—O3B—C6B	14.9 (3)	Cu1—N1B—C5B—C4B	−170.2 (2)
O5A—Cu1—O3B—C6B	−96.53 (19)	C1B—N1B—C5B—C6B	−179.7 (2)
O3A—Cu1—O3B—C6B	110.41 (19)	Cu1—N1B—C5B—C6B	9.8 (3)
N1B—Cu1—O5A—C7A	169.77 (19)	C3B—C4B—C5B—N1B	0.8 (4)
N1A—Cu1—O5A—C7A	−5.67 (19)	C3B—C4B—C5B—C6B	−179.2 (3)
O3B—Cu1—O5A—C7A	−112.23 (19)	Cu1—O3A—C6A—O2A	−179.5 (2)
O5B—Cu1—O5A—C7A	90.25 (19)	Cu1—O3A—C6A—C5A	0.5 (3)
O3A—Cu1—O5A—C7A	−11.0 (3)	N1A—C5A—C6A—O2A	176.8 (2)
N1B—Cu1—O5B—C7B	−5.00 (19)	C4A—C5A—C6A—O2A	−4.1 (4)
N1A—Cu1—O5B—C7B	168.87 (19)	N1A—C5A—C6A—O3A	−3.2 (3)
O3B—Cu1—O5B—C7B	−18.3 (3)	C4A—C5A—C6A—O3A	175.9 (2)
O5A—Cu1—O5B—C7B	91.90 (19)	Cu1—O3B—C6B—O2B	−178.7 (3)
O3A—Cu1—O5B—C7B	−114.98 (19)	Cu1—O3B—C6B—C5B	2.8 (3)
N1B—Cu1—N1A—C5A	140.1 (6)	N1B—C5B—C6B—O2B	173.5 (3)
O3B—Cu1—N1A—C5A	−93.80 (19)	C4B—C5B—C6B—O2B	−6.5 (4)
O5B—Cu1—N1A—C5A	83.18 (19)	N1B—C5B—C6B—O3B	−7.8 (3)
O5A—Cu1—N1A—C5A	179.1 (2)	C4B—C5B—C6B—O3B	172.1 (3)
O3A—Cu1—N1A—C5A	−3.38 (18)	Cu1—O5A—C7A—O4A	−173.7 (2)
N1B—Cu1—N1A—C1A	−34.3 (8)	Cu1—O5A—C7A—C1A	5.5 (3)
O3B—Cu1—N1A—C1A	91.79 (19)	N1A—C1A—C7A—O4A	177.3 (2)
O5B—Cu1—N1A—C1A	−91.23 (19)	C2A—C1A—C7A—O4A	−1.6 (4)
O5A—Cu1—N1A—C1A	4.72 (18)	N1A—C1A—C7A—O5A	−2.0 (3)
O3A—Cu1—N1A—C1A	−177.8 (2)	C2A—C1A—C7A—O5A	179.1 (2)
N1A—Cu1—N1B—C5B	121.0 (6)	Cu1—O5B—C7B—O4B	179.7 (3)
O3B—Cu1—N1B—C5B	−6.50 (19)	Cu1—O5B—C7B—C1B	1.3 (3)
O5B—Cu1—N1B—C5B	179.0 (2)	N1B—C1B—C7B—O4B	−173.3 (3)
O5A—Cu1—N1B—C5B	82.9 (2)	C2B—C1B—C7B—O4B	6.8 (5)
O3A—Cu1—N1B—C5B	−96.7 (2)	N1B—C1B—C7B—O5B	5.2 (4)
N1A—Cu1—N1B—C1B	−49.6 (7)	C2B—C1B—C7B—O5B	−174.7 (3)
O3B—Cu1—N1B—C1B	−177.1 (2)	C9A—N4A—C8A—N6A	−179.6 (3)
O5B—Cu1—N1B—C1B	8.34 (19)	C9A—N4A—C8A—N3A	0.4 (4)
O5A—Cu1—N1B—C1B	−87.8 (2)	C10A—N3A—C8A—N6A	−179.8 (3)
O3A—Cu1—N1B—C1B	92.6 (2)	C10A—N3A—C8A—N4A	0.1 (4)
C5A—N1A—C1A—C2A	1.1 (4)	C8A—N4A—C9A—N7A	−179.7 (3)
Cu1—N1A—C1A—C2A	175.49 (19)	C8A—N4A—C9A—N5A	−1.2 (4)
C5A—N1A—C1A—C7A	−177.9 (2)	C10A—N5A—C9A—N7A	180.0 (3)
Cu1—N1A—C1A—C7A	−3.5 (3)	C10A—N5A—C9A—N4A	1.4 (4)
C5B—N1B—C1B—C2B	−0.7 (4)	C8A—N3A—C10A—N8A	179.5 (3)
Cu1—N1B—C1B—C2B	169.8 (2)	C8A—N3A—C10A—N5A	0.0 (4)
C5B—N1B—C1B—C7B	179.4 (2)	C9A—N5A—C10A—N8A	179.7 (3)
Cu1—N1B—C1B—C7B	−10.0 (3)	C9A—N5A—C10A—N3A	−0.8 (4)
N1A—C1A—C2A—C3A	−0.9 (4)	C10B—N3B—C8B—N8B	−178.3 (2)
C7A—C1A—C2A—C3A	178.0 (2)	C10B—N3B—C8B—N5B	1.1 (4)
N1B—C1B—C2B—C3B	0.0 (4)	C9B—N5B—C8B—N8B	−180.0 (2)
C7B—C1B—C2B—C3B	179.9 (3)	C9B—N5B—C8B—N3B	0.5 (4)
C1A—C2A—C3A—O1A	−179.3 (2)	C10B—N4B—C9B—N7B	−177.6 (2)
C1A—C2A—C3A—C4A	−0.3 (4)	C10B—N4B—C9B—N5B	1.9 (4)
C1B—C2B—C3B—O1B	−179.1 (2)	C8B—N5B—C9B—N4B	−2.2 (4)
C1B—C2B—C3B—C4B	1.2 (4)	C8B—N5B—C9B—N7B	177.4 (2)
O1A—C3A—C4A—C5A	−179.7 (2)	C8B—N3B—C10B—N6B	178.4 (2)
C2A—C3A—C4A—C5A	1.4 (4)	C8B—N3B—C10B—N4B	−1.5 (4)
O1B—C3B—C4B—C5B	178.8 (3)	C9B—N4B—C10B—N3B	0.0 (4)
C2B—C3B—C4B—C5B	−1.5 (4)	C9B—N4B—C10B—N6B	−179.9 (2)

### Hydrogen-bond geometry (Å, °)

**Table:**

*D*—H···*A*	*D*—H	H···*A*	*D*···*A*	*D*—H···*A*
O1A—H1A···O7^i^	0.74 (3)	1.84 (3)	2.573 (3)	169 (4)
O1B—H1B···O6^ii^	0.76 (4)	1.83 (4)	2.579 (3)	173 (4)
N5A—H5A···O10^ii^	0.77 (2)	2.06 (3)	2.787 (3)	159 (3)
N6A—H7···O4B^iii^	0.86	2.13	2.980 (3)	173
N6A—H8···O11	0.86	2.13	2.962 (4)	162
N7A—H11···O1B^iii^	0.86	2.25	3.106 (3)	172
N7A—H12···O9^iv^	0.86	2.11	2.910 (4)	155
N8A—H9···N3A^v^	0.86	2.11	2.973 (4)	177
N8A—H10···O10^ii^	0.86	2.25	2.986 (4)	144
N8A—H10···O11^v^	0.86	2.56	3.202 (4)	133
N5B—H5B···O2A^vi^	0.78 (2)	1.96 (3)	2.698 (3)	157 (3)
N6B—H5···O1A^i^	0.86	2.28	3.131 (3)	170
N6B—H6···O9	0.86	2.38	2.905 (3)	120
N7B—H1···O5B^vii^	0.86	2.24	3.021 (3)	151
N7B—H2···O2A^vi^	0.86	2.11	2.852 (3)	144
N8B—H3···O4A^i^	0.86	2.10	2.930 (3)	163
N8B—H4···O8^vi^	0.86	2.01	2.857 (3)	169
O6—H61···O5A	0.83 (2)	1.87 (2)	2.706 (3)	177 (4)
O6—H62···O2B^viii^	0.81 (2)	1.97 (2)	2.756 (3)	163 (4)
O7—H71···O3B^viii^	0.82 (2)	1.94 (2)	2.741 (3)	163 (4)
O7—H72···O5B	0.82 (2)	1.95 (2)	2.766 (3)	171 (4)
O8—H81···O4B^iii^	0.86 (2)	1.86 (2)	2.714 (3)	173 (4)
O8—H82···O3A	0.83 (2)	1.90 (2)	2.701 (3)	162 (4)
O9—H91···O3B	0.88 (2)	1.97 (2)	2.839 (3)	172 (4)
O9—H92···O4A^iii^	0.88 (2)	2.13 (2)	3.005 (3)	176 (4)
O10—H101···O8^ix^	0.84 (4)	2.24 (3)	3.030 (4)	157 (5)
O10—H102···O4A	0.89 (4)	2.39 (5)	2.695 (3)	101 (4)
O11—H111···O10^x^	0.85 (2)	2.44 (4)	3.196 (5)	149 (7)
O11—H112···N4B^x^	0.83 (6)	2.53 (7)	2.996 (4)	116 (6)
O11—H112···O8	0.83 (6)	2.60 (8)	3.090 (5)	118 (7)

Symmetry codes: (i) *x*, −*y*+3/2, *z*−1/2; (ii) −*x*+1, −*y*+2, −*z*+1; (iii) *x*, *y*, *z*−1; (iv) −*x*+1, −*y*+2, −*z*; (v) −*x*, −*y*+2, −*z*+1; (vi) *x*+1, −*y*+3/2, *z*+1/2; (vii) *x*+1, *y*, *z*; (viii) *x*, *y*, *z*+1; (ix) *x*+1, *y*, *z*+1; (x) *x*−1, *y*, *z*.
